# Medial patellofemoral ligament reconstruction is superior to active rehabilitation in protecting against further patella dislocations

**DOI:** 10.1007/s00167-022-06934-3

**Published:** 2022-03-28

**Authors:** Truls Martin Straume-Næsheim, Per-Henrik Randsborg, Jan Rune Mikaelsen, Asbjørn Årøen

**Affiliations:** 1 Clinic of Orthopaedic Surgery, Akershus University Hospital, 1478 Lørenskog, Norway; 2 Present Address: Oslo Sports Trauma Research Center, Department of Sports Medicine, Norwegian School of Sport Sciences, Oslo, Norway; 3 Institute of Clinical Medicine, University of Oslo, Campus Ahus, Oslo, Norway

**Keywords:** Medial patellofemoral ligament reconstruction, MPFL reconstruction, Patellar dislocation, Conservative treatment, Active rehabilitation, Functional outcome, Adolescence

## Abstract

**Purpose:**

Isolated reconstruction of the medial patellofemoral ligament (MPFL-R) has become the predominant stabilizing procedure in the treatment of recurrent lateral patellar dislocation (LPD). To minimize the risk of re-dislocations, isolated MPFL-R is recommended in patients with no significant trochlea dysplasia and tibial tuberosity trochlear groove distance < 20 mm on computed tomography (CT). Incidentally, these criteria are the same that are used to identify first time LPD patients where conservative treatment is recommended. The purpose of this study was therefore to compare MPFL-R with active rehabilitation for patients with recurrent LPD (RLPD) in absence of the above mentioned underlying anatomical high-risk factors for further patellar dislocations.

**Methods:**

RLPD-patients aged 12–30 without underlying anatomical high-risk factors for further LPD were randomized into treatment either with isolated MPFL-R or active rehabilitation provided and instructed by a physiotherapist. All patients underwent diagnostic arthroscopy for concomitant problems. The main outcome measure was persistent patellar instability at 12 months. Knee function at baseline and 12 months was asses using the following patient reported outcomes measures (PROMS); KOOS, Kujala, Cincinnati knee rating, Lysholm score and Noyes sports activity rating scale.

**Results:**

Between 2010 and 2019, 61 patients were included in the study (MPFL-R, *N* = 30, Controls, *N* = 31). Persistent patellar instability at 12 months was reported by 13 (41.9%) controls, versus 2 (6.7%) in the MPFL-group (RR 6.3 (95% CI 1.5–25.5). No statistically significant differences in activity level were found between the MPFL-group and the Controls at neither baseline nor follow up. The patients with persistent instability at 12 months did not score significantly lower on any of the PROMs compared to their stable peers, regardless of study group.

**Conclusion:**

Patients with recurrent patellar dislocations have a six-fold increased risk of persistent patellar instability if treated with active rehabilitation alone, compared to MPFL-R in combination with active rehabilitation, even in the absence of significant anatomical risk factors. Active rehabilitation of the knee without MPFL-R improves patient reported knee function after one year, but does not protect against persistent patellar instability.

**Level of evidence:**

1.

**Supplementary Information:**

The online version contains supplementary material available at 10.1007/s00167-022-06934-3.

## Introduction

Isolated reconstruction of the medial patellofemoral ligament (MPFL-R) has become the predominant stabilizing procedure in the treatment of lateral patellar dislocation [11]. A plethora of different surgical techniques and a variety of different grafts have been suggested for this procedure; with good functional outcomes and low complication rates [26]. Compared to the bony procedures for stabilizing the patellofemoral joint (i.e. medialisation and/or distalisation of the tuberositas tibia, trochleoplasty, de-rotational osteotomies), MPFL-R is less invasive, possible to perform in patients with open growth plates and seems to include a more benign spectrum of complications [28]. However, patients struggling with recurrent lateral patellar dislocations (RLPD) are a heterogenic group in terms of underlying anatomical risk factors. Studies have shown an increased risk of re-dislocations after isolated MPFL-R in patients with significant trochlear dysplasia (Dejour B-D), patella alta and increased tuberositas tibia trochlear groove (TT-TG) distance [4, 9]. Consequently, in a current concepts review by Weber et al., isolated MPFL-R is recommended in patients with no patella alta, no significant trochlea dysplasia and TT-TG distance < 15 mm on magnetic resonance imaging (MRI) [29]. Incidentally, these criteria are the same that are used to identify patients that are least likely to experience new dislocations after a primary patellar dislocation, i.e., where conservative treatment is recommended [3]. Conservative treatment for primary patellar dislocation has been shown to reduce pain and instability [16, 24].

Previous studies comparing surgery to conservative treatment have generally not accounted for different underlying anatomical risk factors, and only included primary dislocations, where the risk of recurrence is between 25 and 33% [24]. The purpose of this study was therefore to compare MPFL-R with active rehabilitation for patients with RLPD in absence of underlying anatomical high-risk factors for further patellar dislocations. We hypothesized that patients receiving MPFL-R and active rehabilitation would experience fewer episodes of patellar instability and have better patient reported outcome scores one year after surgery, compared with patients undergoing diagnostic arthroscopy and rehabilitation alone.

## Materials and methods

This study was prospective randomized controlled trial comparing MPFL-R with active rehabilitation in patients with RLPD without underlying anatomical high-risk factors for further patellar dislocations.

The study protocol was approved by the Regional Committee of Medical and Health Research Ethics of South East Norway before initiation of the study (REC South East, reference 2009/2148). All patients and their legal guardians (if younger than 18 years) provided oral and written consent before inclusion. The study was registered at ClincialTrials.org (NCT02263807).

### Patients

The study recruited patients referred to the orthopaedic outpatient clinic at Akershus University Hospital for RLPD between May 2010 and January 2019. Patients aged between 12 and 30 years were assessed clinically in addition to plain radiographs and computerized tomography (CT), and invited to participate in the study if they fulfilled the recommended indication for isolated MPFL reconstruction [12, 29] (Table 1). Bilateral cases were excluded as the protocol included functional comparisons with the contralateral leg. Pre-operative Knee MRI was performed to reveal any other concomitant bone, cartilage or soft tissue injury (i.e. ACL-rupture or significant cartilage injury) that would exclude the patient from isolated reconstruction of the MPFL. The Patellar Instability Severity Score (PISS) [3] and Beighton Hypermoibility score [5] were determined for all patients.

**Table 1:** Inclusion criteria for the study based on the recommended indication for isolated MPFL-reconstruction

Inclusion criteria
a. Two or more patella dislocations
b. Positive apprehension test at clinical examination
c. Age 12–30 years
d. Tibal Tuberosity Trochlear Groove (TT-TG) distance < 20 mm on CT
Exclusion criteria
a. Medial dislocation
b. Bilateral patella instability
c. Severe trochlea dysplasia grade D (Dejour)

### Outcome measures

At inclusion, all patients completed these patient reported outcome measures (PROMs):

- KOOS score: the Knee injury and Osteoarthritis Outcome Score is a validated scoring system for assessing outcomes after traumatic knee injury [22], and has also been used for assessing patients after lateral patellar dislocation[2, 15]. Only the “Sport”- and “Quality of life (QoL)”-components were used since these have shown to be most sensitive and relevant for this young patient group [25]. A score above 62.5 for Qol and 75 for Sports has been established as cut off scores for Patient Acceptable Symptom State (PASS) [17].
- Kujala score: the Kujala score is a 13-item questionnaire including different items on pain and instability related to the knee and patellofemoral joint [13].
- Lysholm score: the Lysholm score is a functional score designed for knee ligament injuries, which has also been validated for other knee injuries [27].
- Noyes sports activity rating scale: this Activity scale scores physical activity from 0 to 100 based on sports type and frequency of the activity. It includes a grading of how knee-demanding the activity is from level 1 (High function and demand) to 4 (Sedentary and low demand) [18].
- VAS score: a visual analogue scale from 0 to 10 was used to assess pain
- Modified Cincinnati knee rating system: this is an 8 item questionnaire designed for assessing knee symptoms and function after knee ligament injury on a 0–100 scale [18]. A score above 80 is graded as excellent function [6].

### Surgery and the randomizing process

All included patients underwent a diagnostic arthroscopy to eliminate contra indications and to assess intra-articular pathology. Any meniscal pathology was addressed at the time, and was not a contraindication for inclusion. A case numbered sealed envelope stating either “MPFL” or “Control” was opened per-operatively to randomize the patient to one of the two study groups. These envelopes had been prepared by personnel not involved in the study following a computerized block randomization process (block size 10) before study start.

### Reconstruction group (MPFL)

**Fig. 1:**
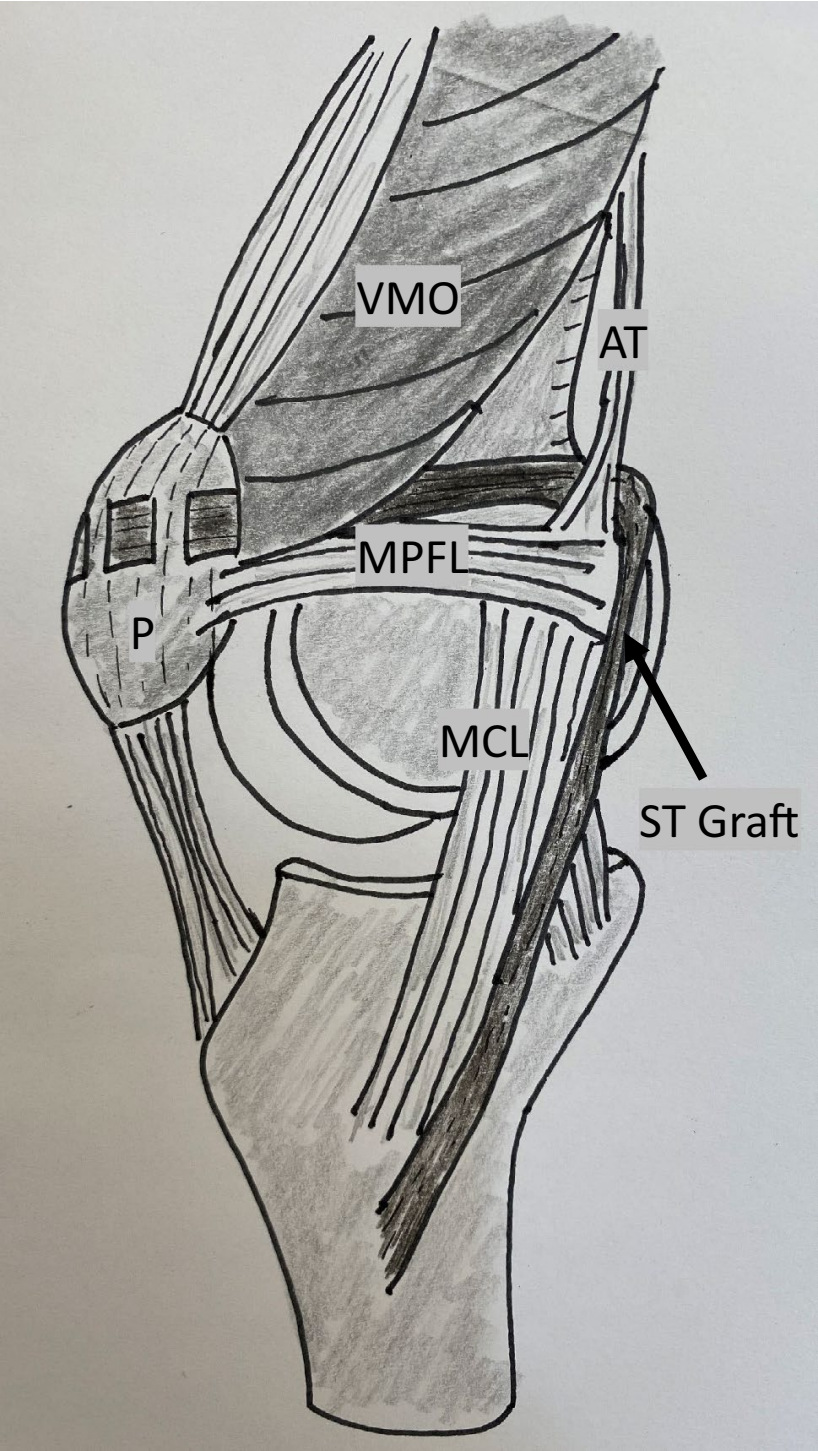
Schematic drawing of the medial patella femoral ligament reconstruction technique modified from Deie et al. [8]. The ST (semitendinosus) graft is left fixed at the tibia insertion, flipped under the adductor tendon and weaved into the periosteum of the patella at 30 degrees of knee flexion. *MCL* medial collateral ligament, *AT* adductor tendon, *MPFL* medial patellofemoral ligament, *P* patella, *VMO* vastus medialis oblique

An open MPFL-reconstruction with a semitendinosus graft according to a procedure modified from Deie et al. [8] was performed (Fig. 1). Semitendinosus was harvested through a standard small incision. The tendon insertion at the pes anserinus was kept. The graft was then transferred to, and flipped around the femoral insertion of the adductor tendon at the adductor tubercle and fixed with two sutures via a small separate incision, before tunnelling the tendon up to the proximal 1/3 of the patella. The graft was weaved and sutured into the periosteum on the anterior of the patella from the medial to lateral while the knee was flexed approximately at 30 degrees. Hence, no bone tunnels were used. This technique ensured that all age groups in the trial could be offered the same surgical treatment. The surgery was performed by at least one of four knee consultants trained in this procedure.

Weight bearing on straight leg was allowed from day one and the patients were instructed to start passive range of motion (ROM) up to 90 degrees of flexion and to practise straight leg raise and quadriceps contractions. After 8 weeks they were instructed to start the same active rehabilitation as the Control group.

### Physiotherapy group (control)

A physiotherapist in our orthopaedic department instructed the patient and provided a home exercise programme and a referral to an external physiotherapist for follow-up (Online Appendix). The training program focused on strengthening of the vastus medialis oblique (VMO), stretching exercises for the hamstrings and neuromuscular balance of the knee. Patellar brace or Mc Connell patellar taping [16] was recommended for the first year in high-risk situations.

### Follow up

All patients visited a designated study physiotherapist 3 and 6 months after arthroscopy/reconstruction to assess pain, ROM, apprehension to lateralization of the patella and to ensure that every patient was followed up by a local physiotherapist. After 12 months the patients underwent a clinical assessment by the surgeon and the PROMs were repeated.

### Statistical analysis

Power calculations were made based on re-dislocations as the main outcome measure. With an assumed percentage of re-dislocations of 30% for the controls and 5% for the MPFL patients [7, 21] the number of patients needed in each group would be 28 given a power of 80% and level of significance of 0.05. Paired or independent *t*-test were used for comparison of normally distributed continuous data, while a Mann–Whitney *U*-test was employed to compare skewed data. Dichotomization using recommended PASS cut off values were used on PROM data to adjust for ceiling effects and skewed data. Binary comparisons were tested using Pearson Chi Square test and odds ratios (OR) were calculated for risk assessment in a multivariate logistic regression model controlling for possible confounders. The level of significance was set to 0.05. All data were analyzed using SPSS version 26 (IBM Corp. Armonk, NY, USA).

## Results

**Fig. 2:**
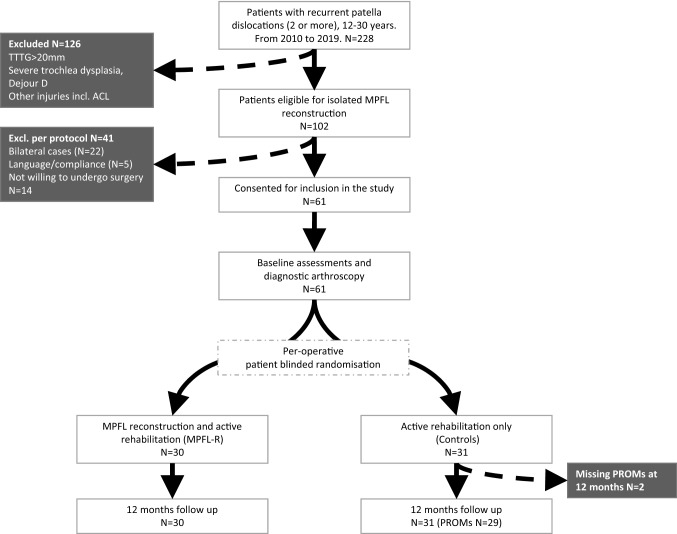
Flow chart of patient selection, randomization and follow up

The study was stopped early by the study oversight group on the basis of a large difference in patient outcomes, and so 61 participants of the originally planned 100 were recruited. These 61 patients were randomized to either arthroscopy and MPFL reconstruction and active rehabilitation (MPFL, *N* = 30) or arthroscopy and active rehabilitation (without MPFL reconstruction) (Controls, *N* = 31) (Fig. 2). Eleven (18%) of the included RLPD patients without anatomical high-risk factors on CT were identified as having a high risk of re-dislocation (PISS ≥ 4), when accounting for their age at their first dislocation and their MRI-findings. These were equally distributed between the two groups (Table 2). The MRI scans revealed 13 (23.2%) cases with TT-TG distance above 16 mm and 7 (11.5%) cases with significant trochlea dysplasia (Dejour B or C). These were found to be evenly distributed between the two groups. No cases of trochlea dysplasia, Dejour type D, were found.

**Table 2:** Demographic comparison of the MPFL-group and Control-group at baseline

	MPFL	Controls
Sex	M 8 (26.7%)	M 9 (29.0%)
	W 22 (73.3%)	W 22 (71.0%)
Age (mean,SD)	18.3 (4.9)	19.9 (5.5)
Under 16 at baseline	11 (36.7%)	11 (35.5%)
Range	12–30	12–30
Side	Left 14 (46.7%)	Left 15 (48.4%)
	Right 16 (53.3%)	Right 16 (51.6%)
BMI (Mean, SD)	21.9 (3.3)	25.3 (5.1)
Range	16.9–31-6	18.8–39.0
Duration of symptoms in months (median)	31.5	29.0
Range	5–184	3–230
Level of activity (%)		
Pivoting sports	13 (43.3%)	13 (43.3%)
No pivoting sports	8 (26.7%)	8 (26.7%)
Less active	4 (13.3%)	1 (3.3%)
Sedentary	5 (16.7%)	8 (26.7)

The arthroscopy revealed a cartilage injury to the patellar surface in 27/61 patients (44%). These were equally distributed between the two groups and all injuries were less than 2 sq cm. A small debridement was performed in only 5 patients (3 Controls and 2 MPFL). No other intraarticular injuries or arthroscopic treatments were performed.

### Persistent patellar instability

At the one-year follow-up, 13 (41.9%) cases in the control group reported persistent patellar instability, versus 2 (6.7%) cases in the MPFL-group (Table 3), which yields a relative risk of persistent patellar instability of 6.3 (95% CI 1.5–25.5) for the control group compared to the MPFL group. This corresponds to an OR of 10.1 (95% CI 2.0–50.2, *p* = 0.005). When controlling for age, gender, Beighton score and activity level, the OR for persistent patellar instability for the Control group compared to the MPFL-R group increased to 19.8 (95% CI (2.9–135.6) *p* = 0.002). Both cases with persistent instability in the MPFL-group were females aged 14 at the time of reconstruction. A PISS-score of 4 or more was significantly associated with persistent patellar instability regardless of intervention [RR = 3.2 (95% CI 1.3–7.5)]. None of the patient in the MPFL-group with a PISS-score under 4 reported persistent patellar instability, compared to 8 (34.8%) patients in the control group.

**Table 3:** Comparison of function between the MPFL-group and the Controls up to 12 months follow up

	MPFL (*N* = 30)	Controls (*N* = 31)	
Persistent patellofemoral instability Max flexion affected leg (degrees)	2 (6.7%)	13 (41.9%)	*p* = 0.005^§^
0 months (mean, 95% CI)	142 (138–146)	136 (132–140)	n.s.*
3 months (mean, 95% CI)	138 (135–142)	138 (135–142)	n.s.*
6 months (mean, 95% CI)	140 (137–145)	140 (137–144)	n.s.*
12 months (mean, 95% CI)	141 (138–145)	140 (137–144)	n.s.*
Positive apprehension (%)			
0 months (*N*, %)	20 (66.7%)	22 (71.0%)	n.s.^§^
3 months (*N*, %)	3 (10%)	7 (22.6%)	n.s. ^§^
6 months (*N*, %)	5 (16.7%)	7 (22.6%)	n.s.^§^
12 months (*N*, %)	5 (16.7%)	12 (38.7%)	*p* = 0.021^§^

*t test,

^§^Pearson Chi-Square

### Symptoms and activity

No statistically significant differences in activity level were found between the MPFL-group and the Controls at neither baseline nor follow up. Almost half of the patients (*N* = 28, 45.9%) reported that their last patellar dislocation prior to inclusion in the study was not sport related. Seventy percent of the patients reported to participate in some type of sports activity on a weekly basis at inclusion, and 43% participated in level 1 pivoting sports like football and handball. Two of six patients who participated in a level 1 pivoting sport daily at baseline had given this up at the one-year follow-up. Both patients were in the MPFL-group, but neither reported any problems with ongoing instability.

**Fig. 3:**
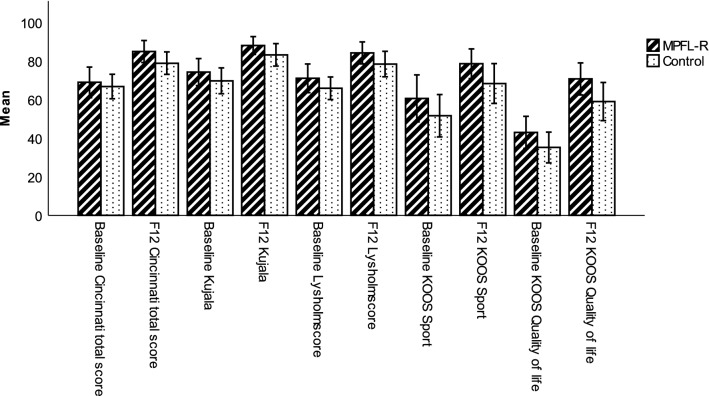
Mean PROMs results (error bars = confidence interval) for the MPFL-R group and Control group at baseline and 12 months follow up (F12). Maximum score for all PROMs was 100 (Excellent function). All improvements from baseline to follow up were significant (paired *t* test, *p* < 0.05), but no significant differences between the two groups were found

For all PROMs, the improvement from baseline to the one-year follow up was statistically significant for both groups (Fig. 3). There was a trend towards better outcome for all parameters in the MPFL-group, but it did not reach the set level of significance. In the MPFL-group, 20 (79%) patients were graded as “Excellent” on the Cincinnati score, compared to 16 (55%) patients in the Control group (*p* = 0.051). The PASS level for the KOOS quality of life score was reached by 22 (79%) patients in the MPFL- group compared to 16 (55%) patients in the Control group (n.s.).

The patients with persistent patellar instability at 12 months did not score significantly lower on any of the PROMs compared to their stable peers, regardless of study group.

### Surgical complications

Anterior knee pain was reported by 6 (20%) of the cases in the MPFL-group and one case reported some diffuse nerve related pain. Only one complication was reported in the control group. This was a female aged 16 at the time of surgery who reported very poor knee function and a preoperative VAS of 8 for pain. She suffered a disabling complex regional pain syndrome in the thigh, most likely caused by compression of the femoral nerve by the tourniquet used under the diagnostic arthroscopy. No difference in ROM was found between the MPFL-group and the Control group at any of the follow-up time points (Table 3).

## Discussion

The main finding in this study is that active rehabilitation without reconstruction of the MPFL gave a six-fold higher risk of persistent patellar instability within 1 year compared to patients who underwent MPFL-R. However, both groups reported significant improvement in PROM scores, indicating that active rehabilitation under instruction of a physiotherapist has a positive effect on knee function irrespective of MPFL-reconstruction and the presence of persistent instability.

Several previous studies have demonstrated that isolated MPFL-R protects well against new lateral patellar dislocations in patients with few predisposing anatomical risk factors for further dislocations [9, 20, 26]. But no previous study has compared surgery with active rehabilitation for this selected group of RLPD-patients. Therefore, until the present study, it was unclear if these good results could be due to the fact that active rehabilitation alone would be sufficient to regain patellofemoral stability in this subgroup of RLPD patients. To that respect, the important take home message from this study is that even for the cases with no significant predisposing anatomical risk factors for further lateral patellar dislocations, active rehabilitation including bracing or taping of the patella did not provide sufficient protection against further patellar instability. In fact, the difference in patellar instability risk between the two groups was getting so obvious that the study group agreed that it was unethical to continue to randomize patents to conservative treatment. The study was therefore stopped after 61 included patients.

As the main criteria for inclusion in the study was two or more lateral patellar dislocations, one might argue that all cases by definition had already failed conservative treatment for their primary dislocation. However, these patients are predominantly young teenagers that are not fully developed. It is likely that most patients had not complied to any proper active rehabilitation after their primary dislocation episode. In addition, the optimal physiotherapy strategy for rehabilitation of these patients is unknown [23]. Among the six most important risk factors for persistent patellofemoral instability, four are pure anatomical features and the remaining two are “bilateral problems” and “age under 16 year at the first episode” [3]. None of these factors can be modified by active rehabilitation. Even after exclusion of the anatomical risk factors, the patients who struggle with RLPD are younger when they experience their first dislocation, they have more often bilateral problems, and as the current study found; they need reconstructive surgery to prevent further instability. This finding is supported by an epidemiological study of patellar dislocations by Fithian et al. [10]. They found that patients with two or more previous dislocations had 7 times higher odds for subsequent instability, but reported no increased occurrence of anatomical risk factors other than a higher lateral overhang measurement indicating a more severe injury to the MPFL and the medial retinaculum.

The study demonstrated a clear difference in persistent patellar instability between the groups, but both groups had less pain and equal improvement in PROM scores from baseline. This demonstrates that rehabilitation, without reconstructing the MPFL, improves pain and patient reported symptoms. Therefore, if pain is the main complaint, conservative treatment is a sensible option, avoiding risk of surgical complications.

The MPFL-R is an appealing extra articular procedure using well-known grafts, and offers a potential quick fix of a complex knee problem that considerably affects the knee function of young patients [25]. From the planning of this study in 2009 to the inclusion of the last patient in 2019, there has been a significant development in the understanding of patellofemoral instability and the role of MPFL-R for this patient group. The pendulum has swung from assuming MPFL-R to be the most crucial procedure in stabilizing the patella, to considering MPFL-R to be an important safety belt after the patella has been put back on track by supplementary bony procedures, or the need of such has been ruled out [4, 9, 29]. In addition, the assessment of the anatomic risk factors for this heterogenic patient group have proven to be difficult, where each component (i.e. patella alta, rotational malalignment, trochlea dysplasia) has multiple assessment strategies and cut off recommendations involving a combination of clinical examination, plain radiographs, CT and MRI [19]. This was reflected when retrospectively reviewing the radiographs and MRIs to score the cases according to the PISS-score published in 2014 [3]. As mentioned, almost 20% was then found to have a high risk of new dislocations, and indeed this score was associated with re-dislocations both for the control cases and the MPFL-R cases. The PISS-score was developed to identify which cases that were likely to re-dislocate after a primary dislocation, but the results from this study suggest that this score (with a cut off at 4 or above) also serves as an indication that supplementary bony procedures have to be considered in patients with recurrent dislocations.

In contrast to previous published studies, this study used a MPFL-R method with no bone tunnels for all age groups. Ironically, the only re-dislocations in the MPFL-group occurred in two skeletal immature patients where supplementary bony procedures and bone tunnel fixations are relatively contraindicated. This emphasize that those who experience their first patellar dislocation before they have skeletal matured have the highest risk of persistent patellar instability, indicating potential multiple underlying risk factors [10]. Our fixation method showed low rates of persistent patellar instability at one year and no significant complications. This finding is supported by a recently published study by Feucht et al. assessing failed isolated MPFL-R [9]. They found that the presence of additional unaddressed anatomical risk factors was the main reason for failure while there was no significant difference between anatomical and non-anatomical femoral tunnel placement. Based on our experience from this study we would hypothesize that the risk of malposition of the femoral tunnel is higher when aiming for an anatomical placement than if a pulley fixation around the adductor tubercle or the posterior MCL is used [8, 14]. According to a review from 2016 the origin of the MPFL appears to be from an area rather than a single point of the medial femoral condyle, and the adductor tendon insertion is within this identified area [1].

The main limitation of this study was the limited cohort size in the final study population. This resulted in a lack of power for the PROMs comparisons, but with a 6 times higher risk of persistent patellar instability in the control group it was concluded as unethical to continue the inclusion up to the planned 100 cases. With only two re-dislocations in the MPFL-group, the confidence intervals for the risk estimates became large. However, the number of events in the MPFL group would have to triple for the risk to become non-statistically significant, indicating that the main conclusion is robust. Furthermore, the follow-up was only 1 year, and it is likely that some patients experienced later re-dislocations. Although, the highest risk for re-dislocations has been reported to be within the first year, the incidence curve does not flatten significantly until after 5 years [11].

Another limitation is that the control group underwent diagnostic arthroscopy, and is therefore not a true conservative (non-operative) control group. Finally, this study recruited patients without significant anatomical risk factors. These criteria applied to under 40% of the patients referred for RLPD [25]. Consequently, our results only apply for the patients in the “mild” spectrum of the heterogeneous population of RLPD-patients.

### Conclusion

Patients with RLPD have a six-fold increased risk of persistent patellar instability if treated with active rehabilitation alone, compared to MPFL-R in combination with active rehabilitation, even in the absence of significant anatomical risk factors. Active rehabilitation of the knee without MPFL-R improves patient reported knee function after 1 year, but does not protect against new dislocations.

## Supplementary Information

Below is the link to the electronic supplementary material.

Supplementary file1 (PDF 625 KB)

## Abbreviations

ACL: Anterior cruciate ligament
BMI: Body Mass Index
CI: Confidence interval
CT: Computerized tomography
KOOS score: The Knee injury and Osteoarthritis Outcome Score
LPD: Lateral patellar dislocation
MPFL-R: Medial patellofemoral ligament
MRI: Magnetic resonance imaging (MRI)
PASS: Patient acceptable symptom state
PISS: Patellar Instability Severity Score
PROM: Patient reported outcome measure
QoL: Quality of Life Score from KOOS
RLPD: Recurrent lateral patellar dislocation
ROM: Range of motion
OR: Odds ratio
Sq cm: Square centimetres
SD: Standard deviation
TT-TG distance: Tuberositas tibia to trochlear groove distance
VAS: Visual analogue scale
VMO: Vastus medialis oblique
