# Supplementary material for: Medial patellofemoral ligament reconstruction is superior to active rehabilitation in protecting against further patella dislocations
Source: Knee Surg Sports Traumatol Arthrosc. 2022 Mar 28;30(10):3428–37. doi: 10.1007/s00167-022-06934-3 (PMC9464184; doi:10.1007/s00167-022-06934-3)
Supplement: Supplementary file 1 — Supplementary file1 (PDF 625 KB) [file 167_2022_6934_MOESM1_ESM.pdf]

Appendix 1
Exercises in the active rehabilitation program for all study patients

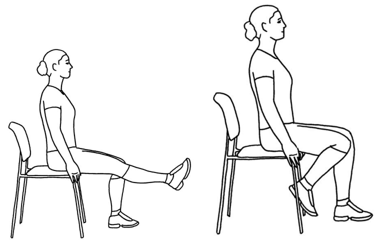
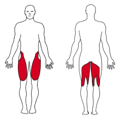

1. Seated knee bending / stretching

Sit on the edge of a chair/bench with both of your feet on the ground. Stretch and bend your knee joint alternately. Make sure that the foot is in contact with the floor throughout the movement.

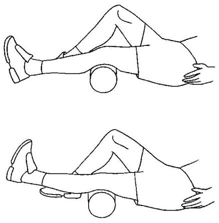
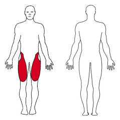

2. Knee Extension, on bolster

Put a bolster, a rolled-up towel or similar under your knee. Rest your ham on the bolster. Flex your front thigh muscles, causing the foot to be lifted off the mat and the knee to stretch maximally. Slowly lower back and repeat the exercise.

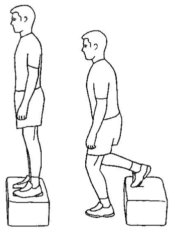
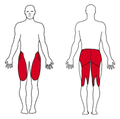

3. Step Down

Stand with your feet hips width apart on the bench/step. Step down from the bench/step alternately with your right and your left leg in front. Perform the exercise slowly and controlled.

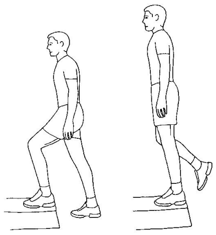
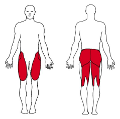

4. Step up on step-bench

Place the active leg on a step-bench, or similar. Lean forward, rest the weight on this leg,and stretch your knee to raise yourself. Gently return to the start position and repeat.

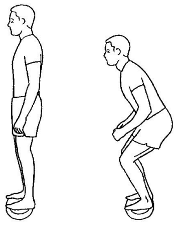
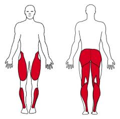

5. Balance pad: Squat

Stand on the balance pad. Bend your knees about 90 degrees and push back up. Keep your back straight and look ahead. You may choose to hold your seated position for a few seconds before pushing back up.

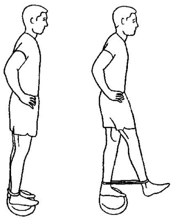
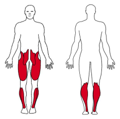

6. Balance pad: Standing forward leg lift w/elastic band

Stand on the balance pad with the elastic band around your ankles. Keep your balance and move your active leg back and forth. Keep your knees straight. Repeat the exercise with your other leg.

Show video

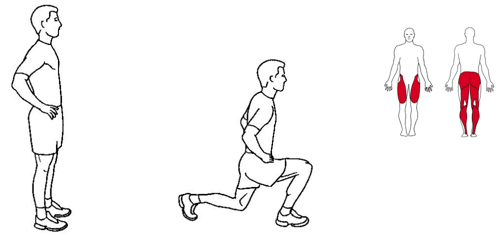

7. Forward lunge

Stand with your legs together and hands by your sides. Raise your active leg and lunge forward. Stop the movement when your foot hits the floor. In the final position your body is in a deep position. Push back up and return to the start position. Repeat with your other leg.

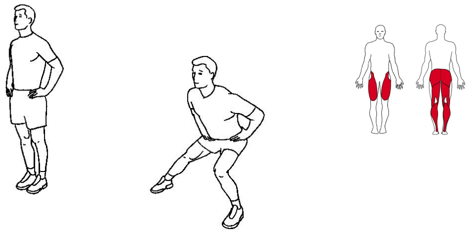

8. Sideward lunge

Stand with your legs together and hands by your sides. Move your active leg sideways and put your weight to that side. Stop the movement when your foot hits the floor. In the final position your active leg is bent and your inactive leg is almost straight. Push off and return to the start position. Repeat to the other side.

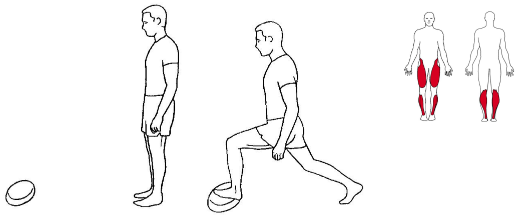

9. Balance pad: Forward lunge

Stand with your legs together, well away from the balance pad. Raise one leg and fall forward toward the balance pad. When your foot hits the balance pad, slow down the movement and stop when your knee bends about 90 degrees and your body is in deep position. Kick away and return to the start position.
